# Immune Checkpoint Inhibitors for Microsatellite Instability High Unresectable Obstructive Colon Cancer: A Report of Two Cases

**DOI:** 10.70352/scrj.cr.25-0070

**Published:** 2025-06-14

**Authors:** Goro Takahashi, Akihisa Matsuda, Takeshi Yamada, Kay Uehara, Seiichi Shinji, Yasuyuki Yokoyama, Takuma Iwai, Toshimitsu Miyasaka, Shintaro Kanaka, Daigo Yoshimori, Takanori Matsui, Koki Hayashi, Hiroshi Yoshida

**Affiliations:** Department of Gastroenterological Surgery, Nippon Medical School Hospital, Tokyo, Japan

**Keywords:** obstructive colon cancer, MSI-H colorectal cancer, pembrolizumab, immune checkpoint inhibitors

## Abstract

**INTRODUCTION:**

Patients with obstructive colon cancer (OCC) with distant metastases often present with a poor general condition, including malnutrition, anemia, and systemic inflammation. Traditionally, these patients undergo stoma creation and/or primary tumor resection followed by systemic chemotherapy. However, for patients with DNA mismatch repair-deficient/microsatellite instability high (dMMR/MSI-H) colorectal cancer, the emergence of immune checkpoint inhibitors (ICIs) has revolutionized treatment strategies, with remarkable antitumor effects. We report two cases of successful management of MSI-H OCC, achieving curative resection while avoiding decompressive procedures, including colostomy creation.

**CASE PRESENTATION:**

**Case 1:** A 29-year-old man diagnosed with MSI-H obstructive transverse colon cancer (cT4b stomach, N1b, M1c1) was treated with pembrolizumab monotherapy (200 mg/body, every 3 weeks). The colorectal obstructive scoring system score was 2 at pembrolizumab administration. The patient showed rapid improvement in his abdominal symptoms within 3 days and achieved clinical complete response after eight courses. Laparoscopic partial colectomy with D3 lymph node dissection was subsequently performed safely. He was discharged on postoperative day 8 without postoperative complications. Histopathological analysis confirmed pathological complete response, and the patient was recurrence-free 15 months after surgery without adjuvant chemotherapy. **Case 2:** A 58-year-old man diagnosed with MSI-H obstructive ascending colon cancer (cT4aN3M1a, LYM) was treated with pembrolizumab monotherapy. The colorectal obstructive scoring system score was 1 at pembrolizumab administration. The patient’s abdominal symptoms improved within 5 days, with marked tumor shrinkage after nine courses. Laparoscopic extended right hemi-colectomy with D3 lymph node dissection was subsequently performed safely, and he was discharged on postoperative day 7 without postoperative complications. Histopathological analysis showed major pathological response (less than 10% viable cancer cells in the resected specimen), with no viable tumor cells in the primary lesion. The patient was recurrence-free 1-year post-surgery, without adjuvant chemotherapy.

**CONCLUSIONS:**

This report highlights the potential benefits of ICI treatment for dMMR/MSI-H OCC, particularly for rapid relief of obstruction-related symptoms and facilitating oncologically safe R0 resection. In cases of MSI-H OCC, ICIs can be highly effective as an alternative to traditional decompression procedures.

## Abbreviations

*BRAF*
: B-Raf Proto-Oncogene, Serine/Threonine Kinase
cCR
: clinical complete response
CT
: computed tomography
dMMR/MSI-H
: mismatch repair-deficient/microsatellite instability high
ICI
: immune checkpoint inhibitor
*KRAS*
: Kirsten Rat Sarcoma Viral Oncogene Homolog
LYM
: para-aortic lymph nodes
mPR
: major pathological response
OCC
: obstructive colon cancer
pCR
: pathological complete response

## INTRODUCTION

Patients with obstructive colon cancer (OCC) with distant metastases usually have poor general conditions, including malnutrition, anemia, and systemic inflammation.^1)^ Therefore, these patients commonly undergo stoma creation and/or primary tumor resection, followed by systemic chemotherapy.^2,3)^ However, in patients with DNA mismatch repair-deficient/microsatellite instability high (dMMR/MSI-H) colorectal cancer, treatment strategies have changed dramatically with the emergence of immune checkpoint inhibitors (ICIs), which have remarkable antitumor effects.^4–7)^ Here, we report two cases of MSI-H OCC in which the obstruction was relieved by ICIs, and safe curative resection was subsequently achieved while avoiding stoma creation.

## CASE PRESENTATION

### Case 1

**Fig. 1:**
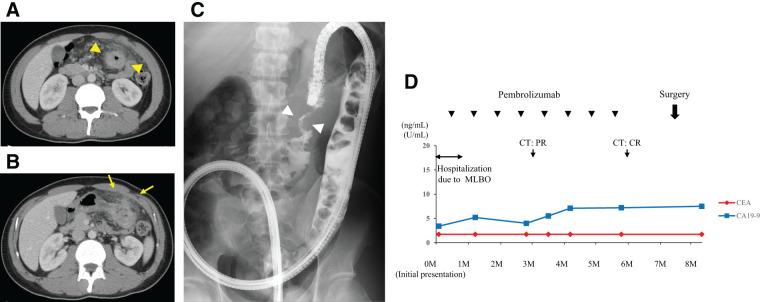
Radiological findings and clinical course in Case 1. (**A**) Circumferential wall thickening of the transverse colon (yellow arrowheads). (**B**) Direct invasion of the transverse colon tumor into the stomach (yellow arrows). (**C**) Severe stenosis was identified by water-soluble contrast medium (white arrowheads). (**D**) Preoperative clinical course. CR, complete response; MLBO, malignant large bowel obstruction; PR, partial response

A 29-year-old man was emergently admitted to our department because of advanced transverse colon cancer with left upper quadrant pain and nausea. Abdominal contrast-enhanced computed tomography (CT) showed thickening of the transverse colon wall contiguous with the stomach wall (**Fig. 1A** and **1B**), local peritoneal dissemination (P1), and regional lymph node enlargement, as well as a marked accumulation of fecal material in the proximal colon. Colonoscopy revealed a circumferential malignant stricture in the left-sided transverse colon, and an approximately 5 cm severe stenosis was identified by water-soluble contrast medium (**Fig. 1C**). Molecular study revealed MSI-H, *KRAS* mutation (G12D), and wild-type *BRAF*. In accordance with the 9th TNM staging system, we diagnosed clinical Stage IVC obstructive transverse colon cancer (cT4b stomach N1bM1c1) with MSI-H.

**Fig. 2:**
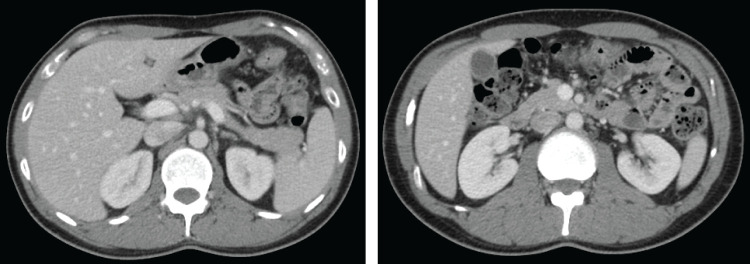
Clinical complete response was confirmed after eight courses of pembrolizumab in Case 1.

**Fig. 3:**
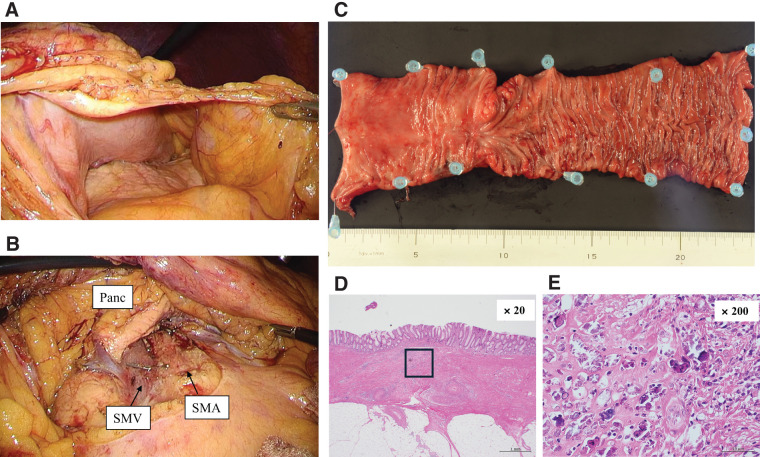
Laparoscopic partial colectomy (transverse colon) with D3 lymph node dissection in Case 1. (**A**) Disappearance of direct tumor invasion into the stomach wall. (**B**) Safe D3 lymph node dissection was possible. (**C**–**E**) Macroscopic (**C**) and histopathological findings ((**D**), (**E**); hematoxylin and eosin) showing complete response. Image (**E**) is a magnified image of the outlined area in image (**D**). Panc, pancreas; SMA, superior mesenteric artery; SMV, superior mesenteric vein

Given the absence of severe abdominal distention (colorectal obstructive scoring system [CROSS] score: 2) and active inflammatory findings, we decided to administer pembrolizumab monotherapy (200 mg/body, every 3 weeks) as first-line therapy under management with only clear liquids orally, without stoma creation (**Fig. 1D**). Three days after administration, the patient’s abdominal symptoms improved, allowing him to continue treatment as an outpatient and resume a normal diet before the second course of chemotherapy. After four cycles, follow-up CT showed a remarkable response in the primary tumor, with no evidence of stomach infiltration. After eight courses (6 months), CT showed clinical complete response (cCR) in the tumor (**Fig. 2**). Subsequently, the patient underwent laparoscopic partial colectomy with D3 lymph node dissection (**Fig. 3A** and **3B**). No postoperative complications were observed, and he was discharged on postoperative day 8. The histopathological findings confirmed pathological complete response (pCR) (ypT0, ypN0 [0/75]); therefore, R0 resection was achieved (**Fig. 3C**–**3E**). Adjuvant chemotherapy was not administered; however, recurrence was not observed 15 months after surgery.

### Case 2

**Fig. 4:**
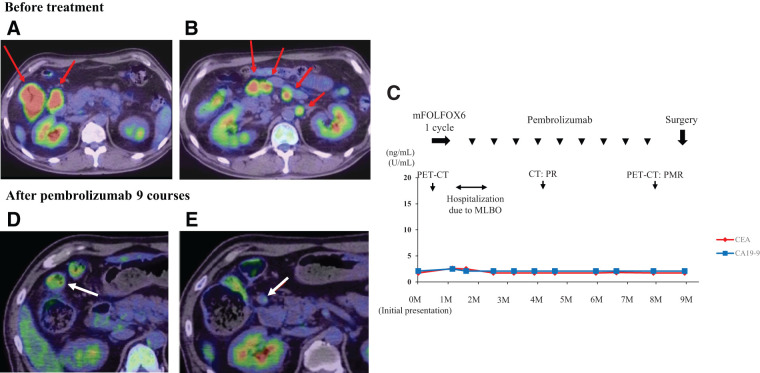
PET-CT findings and clinical course in Case 2. (**A**) and (**B**) Pretreatment images. ^18^F-FDG accumulation is visible in the main tumor, regional lymph nodes, and para-aortic lymph nodes (red arrows). (**C**) Preoperative clinical course. (**D**), (**E**) After nine courses of pembrolizumab, ^18^F-FDG accumulation in the para-aortic lymph nodes disappeared, and only a small degree of ^18^F-FDG accumulation was observed in the primary tumor and one regional lymph node (white arrows). ^18^F-FDG, fluoro-18-deoxyglucose; MLBO, malignant large bowel obstruction; mFOLFOX6, modified oxaliplatin, leucovorin, and 5-fluorouracil; PET-CT, positron emission tomographycomputed tomography; PR, partial response

A 58-year-old man had been diagnosed with locally advanced ascending colon cancer with para-aortic lymph node metastases, staged as cT4aN3M1a (cStage IVA) (**Fig. 4A** and **4B**). Modified oxaliplatin, leucovorin, and 5-fluorouracil (mFOLFOX6) therapy was quickly initiated before confirmation of the molecular study results, which indicated MSI-H, *BRAF* mutation (V600E), and wild-type *KRAS*. However, after one cycle of mFOLFOX6, the patient developed a malignant large bowel obstruction (CROSS score: 1) and was urgently admitted to our department (**Fig. 4C**). After admission, the patient showed slight improvement in abdominal distension with management with no oral intake, alone. Therefore, we continued conservative management and changed the treatment plan from chemotherapy to immunotherapy on the basis of the molecular study results.

**Fig. 5:**
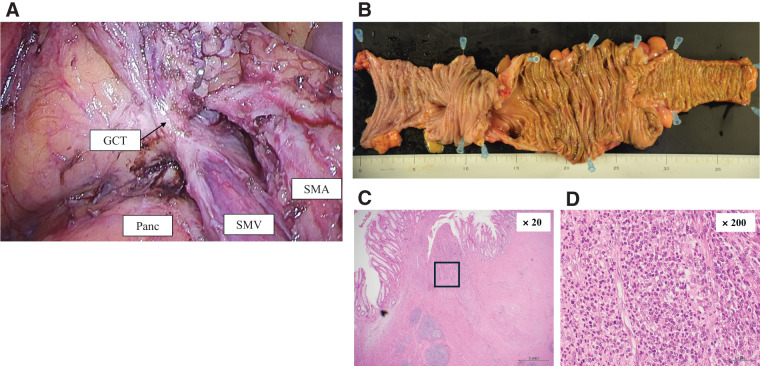
Laparoscopic extended right hemicolectomy with D3 lymph node dissection in Case 2. (**A**) Owing to severe scarring associated with lymph node shrinkage, the GCT was resected at the root to achieve safe complete mesocolic excision. (**B**–**D**) Macroscopic (**B**) and histopathological findings [(**C**), (**D**); hematoxylin and eosin] showing complete response in the primary tumor [image (**D**) is a magnified image of the outlined area in image (**C**)]. GCT, gastrocolic trunk; Panc, pancreas; SMA, superior mesenteric artery; SMV, superior mesenteric vein

Pembrolizumab monotherapy (200 mg/body, every 3 weeks) was administered under clear liquid management. Five days after the start of this therapy, the patient’s abdominal symptoms improved, and he began to have bowel movements. After four cycles, follow-up CT showed a remarkable response in the primary tumor, regional lymph nodes, and para-aortic lymph nodes. After nine courses of pembrolizumab, positron emission tomography CT showed that 18-fluorodeoxyglucose accumulation in the para-aortic lymph nodes had disappeared, with only slight accumulation remaining in the primary lesion and regional lymph nodes (**Fig. 4D** and **4E**). We then performed laparoscopic extended right hemi-colectomy with D3 lymph node dissection. Owing to significant scarring of the regional lymph nodes, we resected the gastrocolic trunk at its root to achieve safe complete mesocolic excision (**Fig. 5A**). The postoperative course was uneventful, and the patient was discharged on postoperative day 7. The pathological outcome was a major pathological response (mPR; <10% viable cancer cells in the resected specimen), with no viable tumor cells remaining in the primary tumor (**Fig. 5B**–**5D**). However, a small number of viable tumor cells were present in one regional lymph node. The final diagnosis was ypT0N1a (1/32) M0, therefore curative resection was achieved. The patient was recurrence-free 1-year post-surgery, without adjuvant chemotherapy.

## DISCUSSION

In this study, we identified two important clinical issues regarding MSI-H OCC. First, ICI treatment rapidly relieved abdominal symptoms related to bowel obstruction, eliminating the need for stoma creation or endoscopic decompression, including stent placement. Second, preoperative ICI treatment facilitated oncologically safe R0 resection.

Pembrolizumab quickly relieved both patients’ obstruction-related abdominal symptoms. In Case 1, improvement was observed by day 3, and in Case 2, by day 5 post-administration. Both patients resumed normal diets by the second course of treatment, while taking magnesium oxide. Additionally, both patients had improved sufficiently in their general condition before surgery, which minimized the risk of perioperative complications.^8)^ This rapid clinical improvement is biologically plausible because a recent phase II clinical trial involving advanced rectal cancer patients (n = 12) showed that viable cancer cells disappeared in biopsy specimens within 6 weeks of ICI administration in the majority of the included patients.^9)^ Although it is important to pay attention to the risk of worsening intestinal obstruction due to phenomena such as hyper-progression and pseudo-progression,^10)^ tumor shrinkage induced by ICI can swiftly relieve obstructions. This makes ICIs an ideal treatment for MSI-H OCC, for which decompression procedures are not as urgent (e.g., CROSS score ≥1).^1)^

In both patients in our report, oncologically safe R0 resection was achieved following eight or nine courses, respectively, of pembrolizumab. Pathological findings showed pCR in Case 1 and mPR in Case 2, with the primary lesion in Case 2 also achieving pCR, and no recurrence was observed over 1 year after surgery. The current Japanese Society for Cancer of the Colon and Rectum Guidelines (2024) recommend pembrolizumab monotherapy as the first-line treatment for unresectable MSI-H colorectal cancer,^11)^ on the basis of the KEYNOTE 177 trial findings.^5)^ This trial reported an overall response rate to pembrolizumab of 43.8%, including a cCR rate of 11.1% (17/153). Although our two cases demonstrated markedly better responses compared with the KEYNOTE 177 results, the reasons behind this discrepancy remain unclear. Recent studies have suggested a possible link between treatment efficacy and the gut microbiome.^12)^ *Fusobacterium nucleatum* (*F. nucleatum*), a Gram-negative anaerobic bacterium, has been reported to increase the abundance of immunosuppressive cells, such as tumor-associated macrophages and myeloid-derived suppressor cells, which play a role in suppressing immune responses within the tumor microenvironment.^13,14)^ By contrast, elevated levels of *F. nucleatum* have also been correlated with improved therapeutic responses to PD-1 blockade in patients with CRC.^15)^ Under conditions of malignant large bowel obstruction, characterized by a particularly anaerobic environment, *F. nucleatum* levels might be further elevated, potentially enhancing the efficacy of ICIs in our cases. Further research is expected to elucidate the detailed mechanisms involved.

Postoperative adjuvant therapy was not administered for both patients. In Case 1, pCR was achieved, and additional treatment was deemed unnecessary due to the uncertain benefit of adjuvant chemotherapy in pCR cases. Conversely, in Case 2, viable tumor cells were identified in local lymph nodes, raising concerns of potential residual disease in the para‑aortic region. While postoperative ICI therapy was initially considered, it was not administered due to uncertainties regarding the optimal duration of adjuvant ICI therapy following a 6‑month neoadjuvant regimen, as well as the risks of mid‑ to long‑term immune‑related adverse events (e.g., colitis, pulmonary toxicity, arthritis).^16)^ Notably, a retrospective analysis in non-small cell lung cancer patients with postoperative recurrence indicated that limited lymph node dissection (LND) was associated with superior ICI efficacy and prognosis compared with systematic LND (2‑year progression-free survival: 45.7% vs. 20.0%, *p* = 0.03).^17)^ These findings suggest that reducing the extent of LND may preserve the local immune microenvironment and lymphatic architecture, which are crucial for lymphocytes trafficking and may enhance ICI effectiveness upon relapse. Accordingly, as our initial surgery spared the para‑aortic lymph nodes, the integrity of lymphatic channels was maintained, which may support a favorable response to subsequent ICI therapy in the event of recurrence.

Initially, we considered self-expanding metallic stent placement for early obstruction relief in both cases, but ultimately decided against this procedure because of the lack of data demonstrating the safety of ICI therapy after stent placement.^18)^ Currently, the Japanese Society for Cancer of the Colon and Rectum Guidelines (2024) do not recommend stenting in patients indicated for chemotherapy.^11)^ By contrast, the European Society of Gastrointestinal Endoscopy Guidelines (2020) suggest that chemotherapy, with antiangiogenic agents (e.g., bevacizumab), can be considered following colonic stenting.^19)^ However, neither of these guidelines addresses the safety of immunotherapy after colonic stenting. Previous representative studies of systemic chemotherapy during self-expanding metallic stent placement reported perforation rates of 2.8%–12.5%.^20–24)^ Therefore, special attention may be needed regarding stent-related perforation during ICI treatment because the antitumor effects of ICIs are more pronounced than those of chemotherapy.

Recently, the “watch and wait” strategy has gained attention, particularly for patients who achieve cCR after ICI administration. This approach involves close monitoring without immediate surgery, aiming to preserve organ function and improve quality of life.^9,25)^ In Case 1, the watch-and-wait strategy was considered an alternative treatment option, but we opted for radical resection because of the still uncertain long-term outcomes of ICI treatment under this strategy. Cercek et al. reported that all 12 patients with advanced rectal cancer treated with dostarlimab in their study achieved cCR and were managed using a watch-and-wait strategy. Remarkably, no recurrences were observed during follow-up periods of 6–25 months.^9)^ Future research should focus on identifying biomarkers, including liquid biopsy,^26)^ that can predict which patients are most likely to benefit from this approach. Additionally, understanding the role of the gut microbiome in modulating the response to ICI treatment could provide valuable insights into optimizing patient selection and improving outcomes.

## CONCLUSIONS

In conclusion, this report highlights the potential benefits of ICI treatment for MSI-H OCC, particularly for rapid relief of obstruction-related symptoms and facilitating oncologically safe R0 resection. The findings suggest that ICI treatment can be an effective alternative to traditional decompression procedures, offering a less invasive option for patients.

## ACKNOWLEDGMENTS

We thank Jane Charbonneau, DVM, from Edanz (https://jp.edanz.com/ac) for editing a draft of this manuscript.

## DECLARATIONS

### Funding

This study received no funding.

### Authors’ contributions

GT and AM drafted the manuscript.

TY, KU, SS, YY, TI, DY, and HY supervised the writing of the manuscript.

GT, AM, TY, and YY performed the laparoscopic surgery.

GT, TMi, SK, TMa, and KH contributed to patient management.

All authors have read and approved the final manuscript.

### Availability of data and materials

The datasets supporting the conclusions of this article are included within the article.

### Ethics approval and consent to participate

Ethics approval for publication of this case report was obtained from the Ethics Committee of Nippon Medical School Hospital. All procedures performed in this study were in accordance with the guidelines of the Declaration of Helsinki.

### Consent for publication

Written informed consent for publication of this case report and the accompanying images were obtained from the patients.

### Competing interests

The authors have no competing interests to declare.
